# 1-Benzyl-1*H*-benzimidazol-2(3*H*)-one

**DOI:** 10.1107/S160053681102455X

**Published:** 2011-06-25

**Authors:** Younes Ouzidan, El Mokhtar Essassi, Santiago V. Luis, Michael Bolte, Lahcen El Ammari

**Affiliations:** a Laboratoire de Chimie Organique Appliquée, Université Sidi Mohamed, Ben Abdallah, Faculté des Sciences et Techniques, Route d’immouzzer, BP 2202 Fès, Morocco; b Laboratoire de Chimie Organique Hétérocyclique URAC21, Faculté des, Sciences, Université Mohammed V-Agdal, Av. Ibn Battouta, BP 1014, Rabat, Morocco; c Departamento de Quimica Inorganica & Organica, ESTCE, Universitat Jaume I, E-12080 Castellon, Spain; d Institut für Anorganische Chemie, J.W. von Goethe-Universität Frankfurt, Max-von-Laue-Strasse 7, 60438 Frankfurt/Main. Germany; e Laboratoire de Chimie du Solide Appliquée, Faculté des Sciences, Université Mohammed V-Agdal, Avenue Ibn Battouta, BP 1014, Rabat, Morocco

## Abstract

The fused five- and six-membered rings in the title compound, C_14_H_12_N_2_O, are essentially planar, the largest deviation from the mean plane being 0.023 (2) Å. The dihedral angle between the benzimidazole mean plane and the phenyl ring is 68.50 (6)°. In the crystal, each mol­ecule is linked to its symmetry equivalent created by a crystallographic inversion center by pairs of N—H⋯O hydrogen bonds, forming inversion dimers.

## Related literature

For the biological activity of benzimidazole derivatives, see: Gravatt *et al.* (1994 ▶); Horton *et al.* (2003 ▶); Kim *et al.* (1996 ▶); Roth *et al.* (1997 ▶). For related structures, see: Ouzidan *et al.* (2011*a* ▶,*b* ▶).
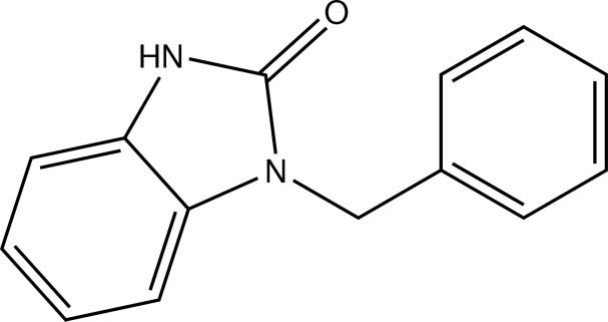


## Experimental

### 

#### Crystal data

C_14_H_12_N_2_O
*M* *_r_* = 224.26
Monoclinic, 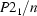

*a* = 13.8652 (7) Å
*b* = 5.7975 (3) Å
*c* = 14.9337 (7) Å
β = 109.5346 (12)°
*V* = 1131.33 (10) Å^3^
*Z* = 4
Mo *K*α radiation
μ = 0.09 mm^−1^
*T* = 298 K
0.50 × 0.44 × 0.28 mm

#### Data collection

Bruker CCD three-circle diffractometer
Absorption correction: multi-scan (*SADABS*; Sheldrick, 1996 ▶) *T*_min_ = 0.959, *T*_max_ = 0.977
9007 measured reflections
3392 independent reflections
2514 reflections with *I* > 2σ(*I*)
*R*_int_ = 0.020

#### Refinement

*R*[*F* ^2^ > 2σ(*F* ^2^)] = 0.045
*wR*(*F* ^2^) = 0.126
*S* = 1.05
3392 reflections
166 parameters
H-atom parameters constrained
Δρ_max_ = 0.21 e Å^−3^
Δρ_min_ = −0.19 e Å^−3^

### 

Data collection: *SMART* (Bruker, 1997 ▶); cell refinement: *SAINT* (Bruker, 1997 ▶); data reduction: *SAINT*; program(s) used to solve structure: *SHELXS97* (Sheldrick, 2008 ▶); program(s) used to refine structure: *SHELXL97* (Sheldrick, 2008 ▶); molecular graphics: *XP* (Sheldrick, 2008 ▶); software used to prepare material for publication: *WinGX* (Farrugia, 1999 ▶).

## Supplementary Material

Crystal structure: contains datablock(s) I, global. DOI: 10.1107/S160053681102455X/im2298sup1.cif

Structure factors: contains datablock(s) I. DOI: 10.1107/S160053681102455X/im2298Isup2.hkl

Supplementary material file. DOI: 10.1107/S160053681102455X/im2298Isup3.cml

Additional supplementary materials:  crystallographic information; 3D view; checkCIF report

## Figures and Tables

**Table 1:** Hydrogen-bond geometry (Å, °)

*D*—H⋯*A*	*D*—H	H⋯*A*	*D*⋯*A*	*D*—H⋯*A*
N1—H1⋯O1^i^	0.86	2.03	2.845 (1)	158

Symmetry code: (i) 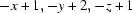.

**Fig. 1.**
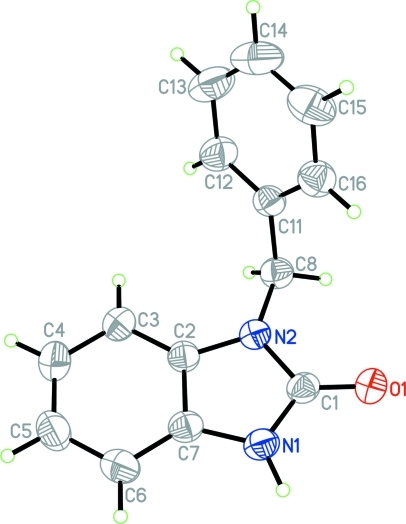
: Molecular view of the title compound. Displacement ellipsoids are drawn at the 50% probability level. H atoms are represented as small circles of arbitrary radii.

**Fig. 2.**
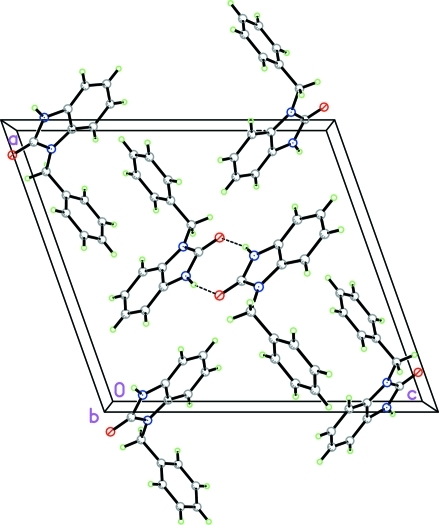
: Formation of pseudo-dimers between two molecules by N–H···O hydrogen bonds (dashed lines).

## supplementary crystallographic information

### Comment

Benzimidazoles are very useful intermediates/subunits for the development of molecules of pharmaceutical or biological interest and its derivatives are an important class of bioactive molecules in the field of drugs and pharmaceuticals. Benzimidazole derivatives have found applications in diverse therapeutic areas including anti-ulcers, anti-hypertensives, anti-virals, anti-fungals, anti-cancers, (Gravatt *et al.* 1994; Horton *et al.* 2003; Kim *et al.* 1996; Roth *et al.* 1997).

As a continuation of our research work devoted to the development of substituted benzimidazol-2-one derivatives (Ouzidan *et al.*, 2011*a*, 2011*b*), we report in this paper the synthesis of a new benzimidazol-2-one derivative by action of benzyl chloride with 1*H*-benzimidazol-2-one in the presence of a catalytic quantity of tetra-n-butylammonium bromide under mild conditions to furnish the title compound (Scheme 1).

The two fused five and six-membered rings are almost planar with the maximum deviation of 0.023 (2) Å from C2. The dihedral angle between the benzimidazole system and the phenyl ring is 68.50 (6)° (Fig.1). In the crystal structure each molecule is linked to its symmetry equivalent created by the crystallographic  inversion center by N–H···O hydrogen bonds to form pseudo-dimers as shown in Fg.2.

### Experimental

To 1*H*-benzimidazol-2-one (0.2 g, 1.5 mmol), potassium carbonate (0.41 g, 3 mmol) and tetra-n-butylammonium bromide (0.05 g, 0.15 mmol) in DMF (15 ml) was added benzyl chloride (0.34 ml, 3 mmol). Stirring was continued at room temperature for 6 h. The salt was removed by filtration and the filtrate concentrated under reduced pressure. The residue was separated by chromatography on a column of silica gel with ethyl acetate/hexane (1/2) as eluent. The compound was recrystallized from ethanol to give colorless crystals (yield: 12%).

### Refinement

H atoms were located in a difference map and treated as riding with C—H = 0.93 Å, and 0.97 Å for aromatic and methylene H atoms, respectively, and with *U*_iso_(H) = 1.2 *U*_eq_(C).

### Crystal data

**Table:**

C_14_H_12_N_2_O	*F*(000) = 472
*M**_r_* = 224.26	*D*_x_ = 1.317 Mg m^−^^3^
Monoclinic, *P*2_1_/*n*	Mo *K*α radiation, λ = 0.71073 Å
Hall symbol: -P 2yn	Cell parameters from 3392 reflections
*a* = 13.8652 (7) Å	θ = 1.7–30.5°
*b* = 5.7975 (3) Å	µ = 0.09 mm^−^^1^
*c* = 14.9337 (7) Å	*T* = 298 K
β = 109.5346 (12)°	Prism, colourless
*V* = 1131.33 (10) Å^3^	0.50 × 0.44 × 0.28 mm
*Z* = 4	

### Data collection

**Table:**

Bruker CCD three-circle diffractometer	3392 independent reflections
Radiation source: fine-focus sealed tube	2514 reflections with *I* > 2σ(*I*)
graphite	*R*_int_ = 0.020
ω scans	θ_max_ = 30.5°, θ_min_ = 1.7°
Absorption correction: multi-scan (*SADABS*; Sheldrick, 1996)	*h* = −18→19
*T*_min_ = 0.959, *T*_max_ = 0.977	*k* = −8→8
9007 measured reflections	*l* = −16→21

### Refinement

**Table:**

Refinement on *F*^2^	Primary atom site location: structure-invariant direct methods
Least-squares matrix: full	Secondary atom site location: difference Fourier map
*R*[*F*^2^ > 2σ(*F*^2^)] = 0.045	Hydrogen site location: inferred from neighbouring sites
*wR*(*F*^2^) = 0.126	H-atom parameters constrained
*S* = 1.05	*w* = 1/[σ^2^(*F*_o_^2^) + (0.0584*P*)^2^ + 0.1866*P*] where *P* = (*F*_o_^2^ + 2*F*_c_^2^)/3
3392 reflections	(Δ/σ)_max_ < 0.001
166 parameters	Δρ_max_ = 0.21 e Å^−^^3^
0 restraints	Δρ_min_ = −0.19 e Å^−^^3^

### Special details

**Table:**

Geometry. All e.s.d.'s (except the e.s.d. in the dihedral angle between two l.s. planes) are estimated using the full covariance matrix. The cell e.s.d.'s are taken into account individually in the estimation of e.s.d.'s in distances, angles and torsion angles; correlations between e.s.d.'s in cell parameters are only used when they are defined by crystal symmetry. An approximate (isotropic) treatment of cell e.s.d.'s is used for estimating e.s.d.'s involving l.s. planes.
Refinement. Refinement of *F*^2^ against all reflections. The weighted *R*-factor *wR* and goodness of fit *S* are based on *F*^2^, conventional *R*-factors *R* are based on *F*, with *F* set to zero for negative *F*^2^. The threshold expression of *F*^2^ > σ(*F*^2^) is used only for calculating *R*-factors(gt) *etc*. and is not relevant to the choice of reflections for refinement. *R*-factors based on *F*^2^ are statistically about twice as large as those based on *F*, and *R*- factors based on all data will be even larger.

### Fractional atomic coordinates and isotropic or equivalent isotropic displacement parameters (Å^2^)

**Table:**

	*x*	*y*	*z*	*U*_iso_*/*U*_eq_	
O1	0.59908 (7)	0.79068 (16)	0.53088 (6)	0.0462 (2)	
N1	0.46509 (7)	0.82117 (17)	0.38695 (7)	0.0391 (2)	
H1	0.4387	0.9497	0.3958	0.066 (5)*	
C1	0.54932 (8)	0.72077 (19)	0.45072 (8)	0.0354 (2)	
N2	0.56888 (7)	0.52373 (16)	0.40851 (7)	0.0350 (2)	
C2	0.49440 (8)	0.49698 (19)	0.31953 (8)	0.0343 (2)	
C3	0.47930 (10)	0.3259 (2)	0.25181 (9)	0.0429 (3)	
H3	0.5232	0.2003	0.2611	0.050 (4)*	
C4	0.39558 (11)	0.3498 (3)	0.16918 (9)	0.0513 (3)	
H4	0.3834	0.2382	0.1220	0.060 (4)*	
C5	0.32981 (10)	0.5368 (3)	0.15565 (9)	0.0510 (3)	
H5	0.2745	0.5477	0.0995	0.058 (4)*	
C6	0.34472 (10)	0.7081 (2)	0.22409 (9)	0.0455 (3)	
H6	0.3002	0.8326	0.2151	0.056 (4)*	
C7	0.42820 (9)	0.68586 (19)	0.30576 (8)	0.0357 (2)	
C8	0.64791 (9)	0.3581 (2)	0.45689 (8)	0.0378 (2)	
H8A	0.6173	0.2060	0.4513	0.044 (4)*	
H8B	0.6744	0.3968	0.5239	0.041 (3)*	
C11	0.73601 (8)	0.34906 (19)	0.41875 (8)	0.0354 (2)	
C12	0.75053 (11)	0.1565 (2)	0.36992 (10)	0.0491 (3)	
H12	0.7048	0.0339	0.3591	0.061 (5)*	
C13	0.83286 (13)	0.1452 (3)	0.33705 (12)	0.0638 (4)	
H13	0.8416	0.0160	0.3036	0.088 (6)*	
C14	0.90122 (13)	0.3235 (3)	0.35368 (12)	0.0659 (4)	
H14	0.9569	0.3145	0.3324	0.085 (6)*	
C15	0.88757 (11)	0.5165 (3)	0.40199 (12)	0.0597 (4)	
H15	0.9338	0.6382	0.4129	0.068 (5)*	
C16	0.80491 (10)	0.5294 (2)	0.43437 (10)	0.0459 (3)	
H16	0.7958	0.6601	0.4668	0.054 (4)*	

### Atomic displacement parameters (Å^2^)

**Table:**

	*U*^11^	*U*^22^	*U*^33^	*U*^12^	*U*^13^	*U*^23^
O1	0.0430 (5)	0.0475 (5)	0.0452 (5)	−0.0005 (4)	0.0110 (4)	−0.0138 (4)
N1	0.0386 (5)	0.0356 (5)	0.0443 (5)	0.0044 (4)	0.0155 (4)	−0.0033 (4)
C1	0.0336 (5)	0.0348 (5)	0.0411 (6)	−0.0025 (4)	0.0171 (5)	−0.0040 (4)
N2	0.0330 (4)	0.0349 (5)	0.0367 (5)	0.0019 (4)	0.0114 (4)	−0.0035 (4)
C2	0.0326 (5)	0.0368 (5)	0.0347 (5)	−0.0011 (4)	0.0130 (4)	0.0000 (4)
C3	0.0447 (6)	0.0405 (6)	0.0429 (6)	0.0018 (5)	0.0137 (5)	−0.0065 (5)
C4	0.0515 (7)	0.0566 (8)	0.0422 (7)	−0.0042 (6)	0.0109 (6)	−0.0116 (6)
C5	0.0412 (6)	0.0656 (9)	0.0404 (6)	−0.0013 (6)	0.0060 (5)	0.0005 (6)
C6	0.0381 (6)	0.0500 (7)	0.0479 (7)	0.0064 (5)	0.0134 (5)	0.0065 (6)
C7	0.0346 (5)	0.0364 (5)	0.0395 (6)	−0.0003 (4)	0.0167 (5)	0.0006 (4)
C8	0.0389 (6)	0.0371 (6)	0.0384 (6)	0.0039 (4)	0.0141 (5)	0.0041 (5)
C11	0.0356 (5)	0.0365 (5)	0.0327 (5)	0.0070 (4)	0.0094 (4)	0.0053 (4)
C12	0.0516 (7)	0.0422 (7)	0.0547 (8)	0.0073 (6)	0.0195 (6)	−0.0022 (6)
C13	0.0697 (10)	0.0637 (9)	0.0675 (9)	0.0226 (8)	0.0354 (8)	−0.0002 (8)
C14	0.0551 (9)	0.0814 (11)	0.0728 (10)	0.0221 (8)	0.0367 (8)	0.0184 (9)
C15	0.0445 (7)	0.0656 (9)	0.0710 (10)	−0.0033 (7)	0.0221 (7)	0.0141 (8)
C16	0.0446 (7)	0.0432 (6)	0.0499 (7)	0.0009 (5)	0.0158 (5)	0.0008 (5)

### Geometric parameters (Å, °)

**Table:**

O1—C1	1.2332 (14)	C6—H6	0.9300
N1—C1	1.3660 (15)	C8—C11	1.5114 (15)
N1—C7	1.3901 (15)	C8—H8A	0.9700
N1—H1	0.8600	C8—H8B	0.9700
C1—N2	1.3749 (14)	C11—C16	1.3824 (17)
N2—C2	1.3922 (14)	C11—C12	1.3845 (17)
N2—C8	1.4546 (14)	C12—C13	1.387 (2)
C2—C3	1.3815 (16)	C12—H12	0.9300
C2—C7	1.3991 (15)	C13—C14	1.368 (3)
C3—C4	1.3897 (19)	C13—H13	0.9300
C3—H3	0.9300	C14—C15	1.379 (2)
C4—C5	1.387 (2)	C14—H14	0.9300
C4—H4	0.9300	C15—C16	1.3869 (19)
C5—C6	1.3902 (19)	C15—H15	0.9300
C5—H5	0.9300	C16—H16	0.9300
C6—C7	1.3783 (17)		
			
C1—N1—C7	110.31 (9)	N1—C7—C2	106.35 (10)
C1—N1—H1	124.8	N2—C8—C11	113.90 (9)
C7—N1—H1	124.8	N2—C8—H8A	108.8
O1—C1—N1	127.38 (11)	C11—C8—H8A	108.8
O1—C1—N2	125.88 (11)	N2—C8—H8B	108.8
N1—C1—N2	106.74 (10)	C11—C8—H8B	108.8
C1—N2—C2	109.46 (9)	H8A—C8—H8B	107.7
C1—N2—C8	123.52 (10)	C16—C11—C12	119.00 (11)
C2—N2—C8	126.56 (9)	C16—C11—C8	120.68 (11)
C3—C2—N2	131.39 (10)	C12—C11—C8	120.30 (11)
C3—C2—C7	121.52 (11)	C11—C12—C13	120.43 (14)
N2—C2—C7	107.09 (9)	C11—C12—H12	119.8
C2—C3—C4	117.10 (11)	C13—C12—H12	119.8
C2—C3—H3	121.5	C14—C13—C12	120.16 (14)
C4—C3—H3	121.5	C14—C13—H13	119.9
C5—C4—C3	121.38 (12)	C12—C13—H13	119.9
C5—C4—H4	119.3	C13—C14—C15	120.03 (14)
C3—C4—H4	119.3	C13—C14—H14	120.0
C4—C5—C6	121.48 (12)	C15—C14—H14	120.0
C4—C5—H5	119.3	C14—C15—C16	120.02 (14)
C6—C5—H5	119.3	C14—C15—H15	120.0
C7—C6—C5	117.27 (11)	C16—C15—H15	120.0
C7—C6—H6	121.4	C11—C16—C15	120.36 (13)
C5—C6—H6	121.4	C11—C16—H16	119.8
C6—C7—N1	132.40 (11)	C15—C16—H16	119.8
C6—C7—C2	121.25 (11)		

### Hydrogen-bond geometry (Å, °)

**Table:**

*D*—H···*A*	*D*—H	H···*A*	*D*···*A*	*D*—H···*A*
N1—H1···O1^i^	0.86	2.03	2.845 (1)	158

Symmetry codes: (i) −*x*+1, −*y*+2, −*z*+1.
